# A history of thalidomide in India

**DOI:** 10.1017/mdh.2023.27

**Published:** 2023-07

**Authors:** Ludger Wimmelbücker, Anita Kar

**Affiliations:** 1 Institute for the History of Medicine and Ethics in Medicine, Charité – University Medicine Berlin, Thielallee 71, 14195 Berlin, Germany; 2 Birth Defects and Childhood Disability Research Centre, Pune 411020, India

**Keywords:** thalidomide, pharmaceuticals, leprosy, India, birth defects

## Abstract

In contrast to the well-known stories of the embryotoxic drug, thalidomide, in countries where it was responsible for large numbers of birth defects, there is limited information on its history in India. Its presence before 2002, when the country issued the first marketing licence for a thalidomide-containing preparation, is assumed to be negligible. This article challenges this view by showing that the drug entered the Indian subcontinent through the former Portuguese territory of Goa around 1960. We examine the subsequent development of its distribution, use and regulation in India from the mid-1960s up to the present situation. Colonial legacies are a crucial explanation for the early appearance of thalidomide on the Indian subcontinent. They also influenced its re-emergence as drug for treating leprosy reactions in India after 1965. We identify key actors in this process: the original German producer that delivered thalidomide free of charge, European doctors who worked for international non-governmental organizations, the World Health Organization (WHO), which supported clinical trials and later discouraged the use of the drug, and finally the Indian state institutions that limited its distribution and later quickly opened the way for the private sector to produce and market thalidomide and its analogues. Finally, we discuss the risk of thalidomide-induced birth defects by casting a critical look on the present state of regulatory provisions and the monitoring of birth defects in India.

## Introduction

The detrimental influence of thalidomide on the early development of the human embryo raised the worldwide awareness of serious adverse effects of drugs during the early 1960s. Thalidomide embryopathy, caused by maternal intake of the drug between days 20 and 36 of gestation, was recognized by the increased incidence of typical limb reduction defects, which were accompanied by additional prenatal damages affecting various inner organs, the eyes and the ears.^1^ Despite well-defined clinical criteria, it is not easy to establish a link between thalidomide and the characteristic anomalies by differential diagnosis. Although the reduction of intermediate parts of the arms, for instance, is a very rare congenital anomaly, it is also a characteristic manifestation of at least twenty-five genetic syndromes. The absence of a family history of limb malformations and a proven history of thalidomide intake still are central criteria in establishing association.^2^ Up to the early 1960s, however, the actual use of the drug was not documented in most cases.

To this day, studies on the history of thalidomide have focussed on the developments that unfolded after the first media reports on the related birth defects appeared in 1961. They usually cover the period from the time when the drug was promoted as a risk-free remedy and extend up to the struggle for compensation in various countries until the early 1970s. Its renewed distribution and use since the mid-1960s, spurred by the interest in its efficacy in managing leprosy reactions, has not been examined in previous studies.^3^ This is the first history of thalidomide in one country from its first appearance around 1960 until the recent past. Reviewing the related developments on the Indian subcontinent that occurred during these six decades is crucial for understanding the evolution of public health regulation of this embryotoxic drug and the unfinished regulatory agenda. Thalidomide has been prescribed for leprosy patients in India since 1966 and gained increasing importance during the last twenty years as a therapeutic in the treatment of multiple myeloma. Central aspects of this history differ fundamentally from the well-known developments in the industrialized countries where thalidomide emerged as a popular hypnotic and sedative around 1960 and was re-approved as a drug from 1998 onwards. In fact, this history is a striking example of the drug’s trajectory in a regional context characterized by the deficiencies of an emerging public health system and the experience of rapidly expanding pharmaceutical markets during recent decades.

This article also presents the first history of thalidomide in India. It focuses on the changes in its distribution and use and the slow development of public health regulations. From a wider perspective, it offers explanations for why the drug did not become widely available on the Indian subcontinent around 1960. We show that European doctors of international faith-based non-governmental organisations (NGOs) as well as the World Health Organization (WHO) played a crucial role in introducing thalidomide as a drug for treating leprosy reactions in the country, which had the largest leprosy population in the world, after 1965. It will emerge that safety concerns were mainly left to the treating doctors and the thalidomide producer until 1986, when the government of India began assuming responsibility for the distribution of the drug to leprosy patients and the monitoring of its adverse effects. The number of approved and off-label uses of thalidomide has grown since its first official registration for the treatment of leprosy reactions and multiple myeloma in India in 2002. We discuss how the changing availability of the drug has influenced the risk of prenatal thalidomide damages. Although there are several pharmaceutical regulatory policies in place, the fundamental challenges of providing the necessary resources for implementing these safety guidelines for embryotoxic substances in a large and diverse country like India still exist till today.

A number of general publications on thalidomide contain information that illustrates the vast range of its international distribution and the occurrence of related birth defects since 1958.^4^ Historical studies, however, largely adopted the national perspectives of those developed capitalist economies in which most of about 5000 surviving babies were born with prenatal damages that were officially recognized as thalidomide embryopathy, nearly 3000 of them in West Germany alone.^5^ In addition, an estimated 3000 to 4000 affected children were born alive but died at an early age.^6^ An unknown number of thalidomide-induced birth defects have not been documented or verified, given the difficulties in assessing thalidomide embryopathy. Birth defects surveillance systems were put in place only after the thalidomide incident. To this day, no confirmed case has been reported from India.

Apart from a number of clinical studies and newspaper articles, there are not many published sources for reconstructing the history of thalidomide on the Indian subcontinent. A large part of our investigation is based on unpublished documents. The archives of the companies that produced thalidomide are generally not accessible to external researchers. The documents collected during the criminal trial against leading representatives of the German thalidomide producer offer important background information as well as some details concerning the early history of the drug in India.^7^ The Israeli State Archives hold the papers of professor Felix Sagher, the superior of Dr Jacob Sheskin who firstly reported the usefulness of thalidomide in the treatment of severe (type 2) leprosy reactions.^8^ The archives of the WHO contain information on in its worldwide use for this indication up to the early 1990s and the role of the original German producer as supplier in this context. We have not been able to access the archival holdings of the Central Leprosy Teaching and Research Institute (CLTRI), Chingleput, or the documents of the Ministry of Health, New Delhi, which contain relevant information, particularly in respect to the last three decades. The hitherto inaccessible records will certainly make it possible to shed light on additional aspects when they are made available to researchers.

Our historical account begins with a look at the ways through which thalidomide entered the Indian subcontinent around 1960. The following two sections examine the re-introduction and the clinical trials of the drug in India following the first report on its efficacy in the treatment of type 2 leprosy reactions. The final section looks at the use of thalidomide after the first official drug safety measures taken in 1986 and the approval of the drug in 2002. The conclusion discusses the risk of thalidomide-induced birth defects in respect to lack of appropriate regulatory practices and absence of a surveillance system for birth defects in India.

## Thalidomide on the Indian subcontinent around 1960

After being synthesised by the pharmaceutical company, Chemie Grünenthal GmbH (Grünenthal), thalidomide was marketed from November 1956 onwards, first in the form of the combination drug, *Grippex*, in West Germany. During the same year, the original producer began searching for international business partners to quickly extend sales of the drug. The most important licencee was the British company, Distillers Company (Biochemicals) Limited (DCBL), which held a production licence for thalidomide as well as sales rights for nearly all Commonwealth countries, including India. The company began introducing drugs containing thalidomide in the home market in 1958. At the same time, the mono-drugs appeared under different brand names, under which they are still presently well known, as for instance *Contergan* in Germany, which was sold under the name *Softenon* in over fifty countries, and *Distaval* in Britain, which was exported under the same name.

Specialized in the production of antibiotics, both Grünenthal and DCBL were eager to extend sales markets during the 1950s and used their international business contacts to export drugs containing thalidomide. Though it seems clear that their interest in entering the Indian market emerged comparatively late, the exact motives for this remain largely unknown since the companies’ archives are not accessible. The available monthly reports of Grünenthal support the idea that the rapid growth of the pharmaceutical industry in India, spurred by foreign investment and import controls after World War II, narrowed sales opportunities for imported pharmaceuticals, even before the Indian Patents Act 1970 reduced the term of granted drug patents to five years and thus laid the foundation for the rapid growth in the production of generics. According to the company’s records, the first antibiotic factory in Southeast Asia, which was built in Pimpri (Pune) with UN support, began quickly increasing the production of antibiotics from August 1955; by June 1957, Indian firms were able to supply the home market with standard antibiotics.^9^

Grünenthal made repeated efforts to access the Indian market from around 1952 and infrequently sold limited quantities of antibiotics in India. The company also submitted a patent specification for thalidomide in 1956 and registered trademarks of drugs containing thalidomide in India.^10^ It examined the possibility of importing *Softenon* in February 1961 and sent a representative to the Indian embassy in May to explore the general opportunities for exports.^11^ However, there is no available evidence showing that thalidomide was exported to India. The situation in the Portuguese colony of Goa was different. Although this comparatively small pharmaceutical market was more developed, the registration of drugs was not required. In December 1956, Grünenthal shipped the ‘first’ order and began monitoring this market with a population of around 600 000 and 250 doctors. In February 1958, the shipments continued and were followed by additional orders of the Portuguese distributor, Drogaria Raicar. In July 1959, this pharmacy expressed interest in introducing Grünenthal’s ‘newest products’, including three preparations containing thalidomide: *Softenon* (the mono-drug), *Noctosediv* (a combination of thalidomide with a barbiturate) and *Poly-gripan* (a remedy against colds).^12^

By November these drugs had been sent to Goa, apparently together with promotional material. As they were available only on prescription, Raicar began training a salesman who specialized in advertising Grünenthal products to doctors in July 1960. The company was generally pleased with the repeated orders and the marketing of its business partner in Goa.^13^ It seems that Grünenthal’s exports came to an end a few months after Indian troops occupied the Portuguese territory on 18 December 1961. Before Goa was integrated as a new state into the Republic of India, other pharmaceutical companies may have also sold thalidomide there, as for instance in the form of the cough medicine, *Peracon Expectorans*, produced by the Germany company, Kali Chemie AG, which was granted a sales licence for Goa by Grünenthal in the middle of 1961.^14^

In contrast to Grünenthal, DCBL was apparently able to establish continuous relations with a local manufacturer or distributor of pharmaceuticals that facilitated the sale of its products in India. A Grünenthal memorandum of talks at DCBL headquarters in London in January 1961 refers to thalidomide sales and states: ‘No sales have been possible in India, because governmental approval has not come through yet. However, they are optimistic that they will receive it and will also be granted the relevant import licenses’.^15^ Presumably, DCBL had delivered samples of drugs containing thalidomide for the purpose of registration in India. It is not unlikely that the company also sent samples to a local business partner. There is, however, no available evidence showing when the necessary permissions were granted, which kinds or which quantities of drugs containing thalidomide actually reached India, or whether samples were given to doctors. Presumably, sales were minimal or, in fact, never commenced until worldwide press reports on the prenatal damages made pharmaceutical companies stop marketing thalidomide in 1961/62.

Away from public attention, and as apparent reaction to the reports on its embryotoxicity, the Ministry of Health listed thalidomide both under poisonous substances and prescription drugs in the amendment to the Drugs Rules of 1945 dated 21 July 1962.^16^ Indian newspapers firstly informed the general public of the possible presence of thalidomide in the country during the second half of August 1962. According to *The Tribune* and *The Pioneer*, the Deputy Health Minister, Dr D. S. Raju, told the Lok Sabha (house of commons) on 24 August 1962 that an importer wanted to import the drug, but the government cancelled the licence because of the toxic effect of the drug recorded from other countries.^17^ The director of the Central Drugs Laboratory in Kolkata, tasked then as now with testing the quality and toxicity of drugs awaiting licencing in India, declared that ‘no consignments had arrived in India’.^18^ On September 15, *The Times of India* maintained that ‘nearly eight drugs’ with chemical structures similar to thalidomide ‘are suspected to be on sale in Bombay and Delhi’ and that doctors had warned the authorities who are aware of the presence of these ‘generics’. On the following day, the newspaper reiterated a Ministry of Health press note saying that thalidomide was not permitted to be imported or marketed in India. The press note mentioned three preparations containing active substances with similar chemical structures, which were known under the trade names *Doriden*, *Megemide* and *Hygroton.* ^19^ The active substances of these three drugs were neither generics nor derivatives of thalidomide but similar chemical compounds that soon turned out not to have the same embryotoxic effect.

On September 17, another article concluded that ‘eight drugs containing radicals of thalidomide […] are on sale in Bombay and Delhi’. It urged the drug control authorities to find out how thalidomide came to be imported, ‘how much of it was imported, and how exactly it was used in processing other sedatives’.^20^ Whether or not authorities followed the final suggestion to send directives to doctors and hospitals cannot be known on the bases of the available evidence. In fact, it is possible that all of these ‘eight drugs’ did not contain thalidomide but similar non-embryotoxic substances because the newspapers did neither mention their brand names nor the designations of their active ingredients. The press reports reveal the uncertainty of both the state authorities and the doctors who were confronted with the question of the embryotoxicity of drugs and set to the task of taking effective measures. This challenge certainly raised concerns not only within the general public but also among the competent authorities and the pharmaceutical industry in India. Significantly, it seems that there were no official warnings mentioning the names of Grünenthal’s or DCBL’s thalidomide preparations. Most likely, even less attention was being paid to the possibility that individual travellers may have taken such drugs to India, not only from Goa but also from West and East Pakistan (Pakistan and Bangladesh), Ceylon (Sri Lanka), Burma (Myanmar), Thailand or other countries, in many of which they were being sold without medical prescription at least until the end of 1961.^21^

While medical publications from this period identify awareness among medical practitioners about the embryotoxic properties of thalidomide, the lack of appropriate official warnings reflect the poor drug regulatory capabilities in the Republic of India at that time. In the 1960s, only a handful of regulations were in place. The Drugs and Cosmetics Act (1940) was the key legislation regulating the import, distribution and manufacture of drugs in the country. Implementation was entrusted with regional state governments, which were limited in terms of manpower and infrastructure to take steps to warn the medical community and public at large about the risks of thalidomide.

Yet, the quantity of the drug that reached India was possibly very small. In the case of Goa, the imports contained a total of about 2.2 kilograms of the active substance.^22^ This figure may indicate that there were very few, if any, surviving children with thalidomide-induced birth defects in Goa, despite the fact that a single tablet can cause such damages.^23^ The main reason for the much less significant presence of thalidomide in the Republic of India was that Grünenthal and DCBL mainly produced antibiotics. The difficulties they faced in accessing the antibiotic market in India, as well as general import restrictions, delayed their efforts to export drugs containing thalidomide. Another reason was the existence of a central drug control authority and the obligation of officially registering imported drugs – in contrast to the former Portuguese territory of Goa, which became part of the Republic of India only a few weeks after the two companies had decided to stop thalidomide sales.

## Leprosy in India and the introduction of thalidomide

The first press reports on Grünenthal’s decision to withdraw thalidomide from the market, which appeared on 27 November 1961, signalled the end of the distribution of the drugs that contained this active ingredient. While sales continued in various countries during the following months, thalidomide emerged as a focus of medical and pharmacological research, both in respect to its embryotoxicity and possible new usages as a vital medicine. The first publication on thalidomide in the treatment of leprosy, submitted by Jacob Sheskin with the support of his superior, Felix Sagher, opened the way for the clinical application of the drug from 1965 onwards.^24^ Its specific effect made it possible to control type 2 leprosy reactions in a high percentage of patients, thus significantly reducing high fever, intense pain and other detrimental conditions, including the nodular skin lesions known as erythema nodosum leprosum (ENL). Thalidomide was more effective in managing these inflammatory episodes than the other drugs that existed in the 1960s. However, it does not reduce the bacillary load and thereby contribute directly to the healing process. The main advantage lies in its immunomodulatory and anti-inflammatory properties, which make it possible to control the autoimmune processes that cause recurrent type 2 leprosy reactions in some leprosy patients.^25^

Although not licensed for use in India, thalidomide was soon used for treating ENL patients. The potential relevance of the drug was particularly high for India since the country reported the largest burden of leprosy cases in the world in the 1960s. The actual number was most likely much higher than the WHO estimate of 2.5 million. In 1991 it was estimated at three million or fifty-four per cent of the world’s total.^26^ The prevalence of lepromatous leprosy (LL) patients with a high bacillary load who were at a higher risk of developing ENL probably ranged below ten per cent. This figure may be taken as an approximate indicator for the occurrence of type 2 leprosy reactions. Therefore, the total number of these cases in India must have been lower than 300 000 or 0.6% of the total population of India, which reached the mark of 500 million at the end of the 1960s. The introduction of the multidrug therapy (MDT) ^27^ in the 1980s not only reduced the overall number of people affected by leprosy but also the occurrence of ENL in treated patients. Globally, it was around 1.2% among all leprosy patients after 1980, although a single Indian study conducted in 1994 reported a prevalence of 0.2%.^28^ Using these data, and keeping in mind that patients may experience multiple episodes of reactions, India possibly had a staggering load of 60 000 to 300 000 ENL cases until the 1990s, when the total number of people affected by leprosy began to decline noticeably. Until that time, the majority of people suffering from ENL in India may have received other modern drugs but not thalidomide.

As a painful and debilitating condition, type 2 leprosy reactions would require some form of treatment or medication. In many cases, the existing medication proved to be effective. Thalidomide, however, certainly was an important addition to the existing cache of medications, especially in the case of patients who did not respond to other drugs. Until the 1950s, the treatment of leprosy and leprosy reactions in India and the Western world was widely based on chaulmoogra oil, extracted from the chaulmoogra tree (*Hydnocarpus wightianus*).^29^ This therapy, together with a detailed and accurate clinical description of leprosy (*kushtha*), was documented in the Sushruta Samhita, a medical text compiled around 600 BC, incorporating traditional knowledge from earlier periods, and even in older ancient Indian texts.^30^ After sulfones had replaced chaulmoogra oil as the key means of healing the disease, corticosteroids as immunosuppressants and clofazimine as antibiotic marked major advances in managing leprosy reactions since the 1960s.^31^ In comparison, thalidomide not only offered more rapid relief and higher efficacy in the treatment of ENL but also seemed to have fewer side effects, except for its embryotoxicity. Clinical studies conducted in the 1960s and 1970s did not establish its known neurotoxic properties in severely affected leprosy patients because it was difficult to distinguish the neurological damage caused by the disease from that caused by thalidomide.

The use of the drug in the treatment of ENL patients spread quickly from 1965 onwards. An important factor in this context was the fact that Grünenthal delivered the drug either free of charge or at the cost price for the purpose of treating type 2 leprosy reactions, thus providing access to the medication both for individual doctors and non-commercial institutions or charitable organizations. This was particularly relevant for India, where leprosy came to be known as an important public health problem during the colonial period. Even though the British government commission of 1891 reported that leprosy appeared to be an insignificant problem, public concern at the ‘imperial danger’ posed by leprosy reaching the British Isles from India led the colonial government to enact the Leprosy Act of 1898. This law (repealed in 1983) institutionalized leprosy patients by gender in order to prevent reproduction.^32^ Care for leprosy patients in the colonial period was available through European doctors and missionary organizations in India.

Faith-based organizations like *The Mission to Lepers (The Leprosy Mission)*, established in 1874, and the *British Empire Leprosy Relief Association* (LEPRA), established in 1924, were joined from the 1950s by similar initiatives to form an extensive network of NGOs providing medical and rehabilitation services to patients. The first truly Indian organization, the *Gandhi Memorial Leprosy Foundation*, was established in Wardha in 1951, in honour of Gandhi’s work to remove stigma and promote inclusion of people afflicted with leprosy.^33^ In 1955, the Government of India launched the National Leprosy Control Programme. In view of the important role of charitable organizations, it is not surprising that thalidomide entered India through related international networks. This evidently applied to leprosy treating centres supported by the *German Leprosy Relief Association* (DAHW), dedicated to addressing the suffering caused by leprosy since its foundation in 1957. This NGO sent a letter to Felix Sagher in 1969 to obtain information on thalidomide. The *Swiss Emmaus* foundation expressed its interest in the drug as early as 1965.^34^

Thalidomide also reached India through international networks of dermatologists. Informal deliveries in this context were not uncommon. In his response to a request from India for a remedy for the severe symptoms of leprosy of a male relative, Professor Felix Sagher wrote in September 1966: ‘Should you need the thalidomide for leprosy reactions, I shall be glad to forward to you a certain amount that could be used under the supervision of your physician’.^35^ While Sagher may actually not have provided the drug in this case, further opportunities for doing so arose during the visit of four colleagues from Bombay during October and November. The neuropathologists, D.K. Dastur and S.C. Divekar, came to Jerusalem to discuss their ‘electrodiagnostical studies’ and meet collaborators, namely Alexander Magora who later published various studies on the neurotoxic effect of thalidomide in leprosy patients.^36^ Like their colleagues before, N.H. Antia, a plastic surgeon, and Sharat C. Desai, a dermatologist and venereologist, participated in the Symposium on Thalidomide in Leprosy at Hansen Hospital, Jerusalem, between 25 October and 1 November 1966. Sometime in 1966–1967 Grünenthal sent 500 thalidomide tablets to Sagher for Desai, who was also present at the second symposium from 2–7 November 1967.^37^

Thalidomide may have reached a hospital supported by *Swiss Emmaus* at the end of 1966. In February 1967, Dr Hans Werner von Schrader-Beielstein, the responsible representative of Grünenthal, advised Dr Lena Wintsch, a Swiss dermatologist at St. Joseph’s Leprosy Hospital in Kankanady, Mangalore (Mangaluru), who had begun using thalidomide and researching its effects: ^38^

The dosage in the treatment of leprosy reactions should be started with 400 mg per patient and per day, divided into 4 times 400 mg.

When the clinical picture improves, the dosage can be reduced immediately and there is no reason to maintain a high dosage in smaller patients.

If your female patients are under control I have no objections to give them thalidomide also in the childbearing age. But please, don’t give women the drug if you are not sure that they will not be pregnant one day. We should be cautious before the scientific research of thalidomide and malformations are not completed and have not come to some conclusions.

During the long-term treatment of chronic lepra patients in Israel – they didn’t get any drug at all except thalidomide for 1 year and longer – there were no peripheral neuritis which could be attributed to the drug.

By 1967, there was little doubt about the embryotoxic properties of thalidomide in humans since tests had shown similar birth defects in various animal species. At the same time, it was an unresolved question to what extent the neurotoxic effects, which were already known before 1962, would also appear in leprosy patients under thalidomide therapy.

Responding to von Schrader, Dr Wintsch did not come back to this issue but highlighted the success in treating leprosy reactions in April 1967. She reported that it had been possible to stabilize the patients and withdraw steroids, namely to replace ‘Prednisolone and raise the doses of anti-leprosy drugs’.^39^ Thus, it was possible to reduce the various side effects of steroids, as for instance the risk of infection caused by their immunosuppressive effect. In 1968, Wintsch wrote up a report on the positive results of her research in India and continued treating leprosy patients with thalidomide after returning to Switzerland.^40^

In the above-cited letter of April 1967, Wintsch asked to recieve another ‘package of thalidomide’ as soon as possible and mentioned that she was ready to cover the air mail charges. She added: ‘If you just declare it as anti-leprosy drug and gift, it will arrive safely’. This statement indicates that she received the previous supply either during a home leave or through a visitor from overseas, in fact without informing the competent Indian state authorities. In August 1968, the superintendent of St. Joseph’s Leprosy Hospital, Dr Macaden, asked von Schrader for additional thalidomide supplies. With the apparent intention of delegating the responsibility for the distribution and use of the drug, Grünenthal sent 3000 tablets free of charge to the Emmaus foundation in Switzerland.^41^ The informal way in which thalidomide made its way to Mangalore shows how the drug must have frequently entered India from 1966 onwards.

There were also individual doctors in India who tried to get thalidomide for their ENL patients, as for instance Dr Désiré Rebello in Bombay. After writing a letter to Sagher in Jerusalem, who forwarded it to von Schrader, Grünenthal immediately sent her 500 thalidomide tablets in December 1967. Because the customs authority did not release the parcel, von Schrader wrote a letter to the Drugs Controller, Directorate General of Health Services, New Delhi, not only attaching literature on the use of thalidomide in the treatment of leprosy reactions but also mentioning that the WHO had begun conducting tests of the drug in various countries, including India. Thus, Dr Rebello received the drug and gave it to some of her patients.^42^ It seems that she did not continue using thalidomide over a long period since she did not inform Sagher about the results nor respond to a questionnaire sent to her in 1972, presumably because she did not have the resources to fully participate in the network of doctors using and testing thalidomide.^43^

Not all the doctors showed an interest in the new remedy in the same way as those who tried to order it. Dr Karat, the superintendent of the Schieffelin Leprosy Research Sanatorium, Karigiri, Vellore, which was being supported by The Leprosy Mission (UK) and American Leprosy Missions (USA), wrote in 1967:^44^

Thalidomide is not available in India, neither is there any possibility of its being distributed indiscriminately throughout the country as a panacea for the ills of leprosy. […]

I must re-emphasise that none of us envisage the use of Thalidomide as a specific anti-leprosy drug. Probably you are aware that in the natural history of this disease 20 to 50% of patients pass through a phase of what is commonly referred to as ‘reaction’. The majority of these patients can be controlled with simple anti-inflammatory drugs and some of them require Prednisolone. There is a small percentage of patients who enter the sub-acute and chronic phase of exacerbation which renders them incapable of being treated with specific anti-leprosy drugs. This is one of the dilemmas in leprosy – namely, exacerbation of the disease following administration of specific drug. It is in this very small group of patients that one sees a place for the use of Thalidomide as a temporary measure, to enable these patients to get over the period of recurrent and chronic reactive phase and render them capable of tolerating specific anti-leprosy drugs.’

This cautious approach was shared by Dr C.G.S. Iyer, the director of the Central Leprosy Teaching and Research Institute (CLTRI), Chingleput, a national institute that started as a missionary leprosy sanatorium in 1924, was taken over by the Government of India in 1955 and has functioned as a key training and research institution of the Directorate General of Health Service since 1974.^45^ In January 1967, von Schrader sent 1000 tablets to Iyer, apparently on his own initiative. The cover letter highlighted that the ‘tablets should be used for the treatment of leprosy reactions only’. Von Schrader also enclosed a letter from the WHO office in Geneva as legitimization for the import of the same quantity of tablets. The available information suggests that Iyer neither requested these tablets nor used them.^46^

This incidence shows that Grünenthal was keenly interested in extending the new use of thalidomide and involving official institutions in the emerging network of thalidomide users. Imports into India were possible in the form of ‘gifts’, by attaching suitable cover letters, and also by conveying them in the personal luggage of travellers. The responsibility for the distribution and use of the unapproved drug was less with the company than with the doctors who prescribed the drug, a condition that did not change much until 1985.

## Clinical trials supported by WHO and Grünenthal

The continuous supply of thalidomide from Germany made it possible for the WHO to launch the multinational double-blind trial that was conducted at leprosy treating centres in Spain, Mali, Somalia and India between 1967 and 1970. The Indian trial, which actually commenced in March 1968, was carried out by Iyer and other investigators of the CLTRI. The drug manufacturer was instructed to produce identical tablets containing either thalidomide or acetylsalicylic acid and send them to the four centres. By focusing on male patients only, the trial excluded women participants – just like nearly all the clinical studies on thalidomide that had been published so far, while in reality the drug had also been given to women patients right from the start. The drug treatment intervals were limited to seven days, thus reducing the risk of thalidomide-induced neuropathy. The final report concluded that ‘thalidomide is consistently superior’ compared to acetylsalicylic acid. It mentioned embryotoxicity and leucopoenia (reduction of white blood cells) as side-effects of thalidomide, but it more clearly pointed out the risk of permanent nerve damage considering the fact that the ‘early detection and differential diagnosis of drug-induced neuritis has its specific difficulties in leprosy patients because of the neuritis caused by *Mycobacterium leprae*’.^47^

The investigation at CLTRI was the first study on thalidomide in India that became widely known in the country. Its high profile as WHO multinational research and the continuation of additional trials at this state institute certainly contributed to legitimizing the use of thalidomide among Indian doctors. However, the generally positive study report that appeared in 1971 had no immediate influence on the use of thalidomide in India. At the end of 1972, Sagher and Sheskin sent a questionnaire to leprosy treating centres worldwide to collect corresponding information. Out of sixteen doctors practising in India, six did not respond and seven answered negatively. Three of these respondents were of the opinion that the use of thalidomide was prohibited in India.^48^ Only the remaining three, including the above-mentioned Dr Wintsch, provided information on the use of thalidomide in the treatment of leprosy reactions.

In response to the questionnaire, Dr Elisabeth Vomstein reported that thalidomide had been in use at the Leprosy Rural Relief Center, Chettipatty, since 1970. It was given to six male lepromatous leprosy patients for a period of 18 months. A daily dose of 200mg had sufficed to control the leprosy reactions up to three to four weeks. It was possible to reduce the maintenance dose to 100mg and 50mg but not to completely withdraw it. Combined with sulfones (DDS) and other anti-leprosy drugs, the therapeutic outcomes were classified as ‘satisfactory’.^49^ According to Dr Samuel Pickens of the Christian Fellowship Hospital in Ambilikkai, Tamil Nadu, the treatment of leprosy reaction began in 1972. There was no woman of childbearing age among the nine patients. Usually applied together with an unspecified anti-leprosy therapy for some weeks, initial daily doses of thalidomide varying between 200–400mg followed by a reduced dose of 100mg produced little side effects (‘somnolence’, ‘skin eruptions’). The results were reported as ‘excellent’ in six lepromatous leprosy patients and ‘satisfactory’ in three borderline cases.^50^

While the number of patients treated with thalidomide was on the rise from about 1973 onwards, drug safety measures seem to have decreased temporarily. This was the case with two published trials of Isoprodian, a drug that was a combination of prothionamide, isoniazid and dapsone, with or without rifampicin; thalidomide was used for controlling leprosy reactions during the therapy.^51^ In the trial that began at Leprosy Rural Relief Centre in May 1973, Vomstein included three young women among the total of thirty-two patients. She pointed out that twenty-eight of these participants needed to be treated with thalidomide to manage the reactions.^52^ In 1974, Maria Aschhoff enrolled thirty-eight patients, including thirteen women, in a trial conducted at St Thomas Hospital in Chetput. The majority of the patients required treatment with thalidomide. It is noteworthy that Aschhoff reported one female patient who ‘withdrew from the trial on her own initiative’ after she had an abortion. Three patients ‘had to be withdrawn from the trial on account of repeated severe reactions resulting in paralysis’.^53^ The fact that thalidomide is associated with increased risk of miscarriages and can lead to nerve damage was not discussed by the authors.

Except for the CLTRI, which participated in the international study supported by the WHO, the publications on the use of thalidomide in India were authored by European doctors working for foreign NGOs until the beginning of the 1970s. However, the address list of thalidomide recipients compiled by Grünenthal in 1972 also includes Indian doctors, viz. Professor A.J. Selvapandaram of the Christian Medical College Hospital in Vellore, Dr K. Sathyananrayana at the Central Leprosarium in Bangalore and Dr S.J. Irani at the Sir J.J. Hospital in Bombay.^54^ There certainly were more Indian doctors who received thalidomide, either directly from the producer or through international NGO networks. Because Grünenthal only sent a limited quantity to India, the drug must have become part of the regular therapy of leprosy reactions in some but certainly not in a large number of hospitals at that time.

The first publications on thalidomide trials authored by Indian doctors appeared during the 1970s. They all originated from the CLTRI and were again carried out on behest of the WHO, which supported a number of anti-leprosy drug studies in different countries at that time. Their main objective was to examine clofazimine as a means of reducing the dose of sulfones (DDS) and prevent leprosy reactions in order to find an alternative for patients dependent on the long-term prescription of steroids. Thalidomide was either used as control drug or compared with clofazimine, which proved to be effective in controlling leprosy reactions, or combined with clofazimine and tested as monodrug.^55^ It seems that between 50 and 100 male patients took thalidomide during the trials at the CLTRI, which started in 1973. Around the same time, Grünenthal established the general practice of asking the recipients to sign a declaration of commitment before delivering the required quantity of thalidomide tablets. This was evidently the case for the institute director and principal investigator, Dr Iyer, who, as reported in 1975, had delivered a ‘written statement that he accepts full legal and medical responsibility for the use of the drug […] in respect to his ongoing research’.^56^

The investigators working for non-governmental hospitals, who probably were required to also sign this declaration, followed the same line of research. One study concluded:

To minimize steroid usage, thalidomide and clofazimine combination can effectively control type II lepra reaction, with minimal side effects. Hence we believe that thalidomide should be freely available. If the competence of staff using this drug is enhanced, morbidity due to leprosy can considerably be reduced.

In a sceptical comment, the editor recalled the risk for the unborn child.^57^ Additional thalidomide trials conducted at two government hospitals, the Dr VM Medical College in Solapur and the Dr Bandorwalla Leprosy Hospital in Pune, yet again arrived at positive results in treating ENL patients.^58^ Altogether, more than 200 test persons participated in the trials mentioned in this paragraph, but apparently no female patients.^59^

While the use of thalidomide was a contentious issue and its distribution not officially encouraged, it is likely that the great majority of Indian doctors preferred to use more easily available drugs and not normally prescribe thalidomide to women of childbearing age. The restraint towards the drug also corresponds to the fact that there were no publications in India on thalidomide trials for other indications than ENL up to the 1990s. Thalidomide was frequently used as the last line of treatment the case of type 2 leprosy reactions. The doctors at CLTRI were even convinced that ‘the control of severe reaction is not as efficient as with corticosteroids and therefore it cannot replace steroid in severe reaction’. Despite their adverse effects, chloroquine, isoniazid (INH) and sulfones were given to patients with recurrent or chronic leprosy reactions. Thalidomide and clofazimine were the remedies of choice only in the case of ‘repeated steroid dependant’ reactions. There were different kinds of therapy for specific complications such as ‘acute painful neuritis’, ‘acute eye manifestation’ or ‘arthritis and periostitis’.^60^ The choice of this line of treatment was possibly influenced by the cautious policy of the Ministry of Health towards thalidomide.

The early active role of the WHO in supporting the testing of the drug found an end with the stance of its Expert Committee on Leprosy, which has not recommended the general use of the drug since 1970.^61^ Likewise, the Indian state authorities did not actively promote the use of thalidomide nor support its distribution. Without this backing, it was up to the individual doctor to take up responsibility for its administration and for any serious side effects. An alternative was to treat the majority of ENL patients with steroids, normally used in addition to clofazimine as antibiotic. This combination acts much more slowly but, in contrast to thalidomide, contributes to reducing the bacillary load and with it the occurrence of leprosy reactions. This line of medication conformed with the government’s leprosy eradication programmes. The quantities of thalidomide that reached India were low considering the high number of potential patients. For the great majority of people affected by leprosy, access to thalidomide depended on private medical providers for whom it was not always an easy option to obtain the drug from Germany.

International NGOs continued to play a crucial role in this respect since they were the main providers of services for leprosy patients. Among them was Swiss Emmaus foundation, which not only supported the St Thomas Hospital and Leprosy Centre in Chetput but also the St John’s Leprosy Hospital in Mangalore and the Hubli Hospital for the Handicapped in Karnataka. The last two requested thalidomide directly from Grünenthal sometime between August 1984 and January 1985. This also holds for the Leprosy Mission in New Delhi and the Vimala Dermatological Centre in Bombay (established by the missionaries of the Immaculate Conception). Besides the DAHW, the German Institute for Medical Mission and the Diakonisches Werk (Germany) also ordered supplies for treating leprosy reactions, a part of which they seem to have forwarded to mission hospitals in India. Another possible source apart from these three German charitable institutions was the Sovereign Order of Malta in Geneva. A government medical college at Rohtak also requested thalidomide from Grünenthal.^62^ The Tuberculosis Research Centre in Chennai placed an order with the WHO in June 1985 to treat ENL patients, but the drug was probably not delivered this way.^63^

All these details show that the free supply by Grünenthal continued to be the driver behind the increasing use of thalidomide as a remedy for leprosy reactions in India. German-speaking doctors employed in the country were early actors in this process. In the absence of any official regulation, the German distributor took the initiative in establishing the first general safeguards for the use of the embryotoxic drug in the mid-1970s by asking the recipients to sign a declaration and take up formal responsibility for its control. The government of India began implementing general safety measures only after the German producer decided to discontinue the extant distribution practice of thalidomide in 1984, as we shall see in the following section.

## From distribution control to the free market

There was no general authorization for the distribution and use of thalidomide in India during the four decades that followed its ban promulgated in 1962. Grünenthal did not apply for the official registration of the drug but informed the Drugs Controller about its importation for the first time in 1968. Hospitals and individual doctors were able to obtain thalidomide by way of an import licence up to 1984.^64^ In the middle of this year, Grünenthal stopped the supply of thalidomide and asked the WHO to take over distribution of the drug. The company’s apparent intention was not only to avoid any adverse publicity and litigation but also to limit its production and global distribution. When negotiations with WHO in 1985–1986 did not lead to the desired result, the company required national governments to take up the responsibility for any liabilities emerging from the possible occurrence of any serious side effects and asked them to select one institution as recipient and national distributor. The Directorate General of Health Services, New Delhi, signed the corresponding ‘Agreement concerning the distribution of Thalidomide’ on 18 February 1987.^65^

First in 1986, the Ministry of Health nominated the director of CLTRI ‘as the nodal officer for distribution of Thalidomide tablets to different institutions/organisations in the country involved in treatment of reactions in leprosy patients’. It also stipulated that ‘the CLTRI will ensure prompt supply of the drug after making sure of the indications as well as contra indications for the drug’. Finally, the ministry placed an order of 300 000 thalidomide tablets (100mg) with the WHO.^66^ One third of these were actually delivered to the CLTRI. In 1991, the institute director informed Grünenthal that 100 000 tablets had been utilized with ‘no adverse reaction whatsoever […] reported uptill now’ and added that the Directorate General of Health Services is going to request the same quantity ‘with a fresh agreement form’.^67^ The Director of Health Services placed the order soon afterwards. In the next letter sent to the company two years later, he pointed out that the ‘tablets have been used under supervision of the trained medical officers in the field and have given excellent results’. He added that no side effects had been reported and asked the company to send another 200 000 tablets to the CLTRI.^68^

The 100 000 tablets used between 1986 and 1991 allowed for treating not much more than 1000 patients. This means that thalidomide probably reached less than one per cent of the estimated total number of potential ENL patients in India who might have profited from taking it. It seems that the imported quantities did not suffice to continue the treatment of type 2 leprosy reactions on the previous level. They made it almost impossible to conduct additional clinical studies or use the drug for other indications in India after 1984. Following reports on the interruption of thalidomide supplies, the Indian Association of Leprologists warned about ‘the grave situation in the treatment of leprosy reaction faced by several projects in India’ and added:

We have several patients improving remarkably with this drug and we face the consequences of recurrence of ENL if the patients are denied the benefit of maintenance dose. Further many more cases where there is a scientific [sic] indication for Thalidomide will be denied the benefit of this drug.^69^

The president of the Poona District Leprosy Committee, Dr Jal Mehta, was eager to acquire additional supplies after Grünenthal had begun sending consignments to the CLTRI. In February 1987 he complained that a Brazilian trading company charged a price fifteen times higher than Grünenthal. Four years later he criticized the bureaucratic obstacles and the permitted quantities as insufficient for the treatment of the local ENL patients.^70^ At least temporarily, hospitals funded by foreign NGOs may have been in a better position to tap the drug from external sources than locally funded institutions.^71^ Others would have to drastically reduce its use and make do with irregular supplies, as for instance the Bombay Leprosy Project that received tablets from a colleague in Spain.^72^ In diametric contrast to the thalidomide production and distribution by the government of Brazil that began in 1966, the availability of the drug in India was limited, in particular between 1985 and 2002 when supplies hardly allowed for conducting any clinical trials.^73^ Indian experts later rued this situation and the lost opportunity for providing relief to patients with ENL in the country.^74^

Globally, the evidence for the immunomodulatory and anti-inflammatory properties of thalidomide led to an increasing number of clinical trials that tested its efficacy for difficult-to-treat conditions since the 1980s. These conditions included host-versus-graft disease, rheumatoid arthritis, systemic lupus erythematosus, Bechet’s disease, AIDS-related Kaposi’s sarcoma, Crohn’s disease and psoriasis.^75^ The commercial potential of thalidomide came to the forefront with reports of its efficacy in the management of chemotherapy refractive or resistant multiple myeloma, the second most common haematological malignancy. Reports of its antiangiogenic properties in 1994,^76^ were rapidly followed up by clinical trials in 1999 that demonstrated high response rates, improved remission duration and increased survival of patients with refractive or resistant multiple myeloma.^77^

These clinical studies were made possible by more frequent supplies of thalidomide by Grünenthal for other indications than leprosy and its additional distribution by companies such as Celgene, New Jersey, USA. From 2000, Grünenthal provided the drug free of charge in support of a series of multiple myeloma trials at the Rotary Cancer Hospital, All India Institute of Medical Sciences (AIIMS), a premier public teaching hospital in New Delhi. The first reported trial tested the tolerability of high doses of thalidomide (800mg/day) for a prolonged period of twelve to fourteen months.^78^ The investigators concluded that the drug was safe for long term use, with minimal side-effects that did not warrant stopping of therapy, despite the fact that peripheral neuropathy prevalence was at thirteen per cent. The investigators obtained informed consent from trial participants according to WHO guidelines and the requirements of Grünenthal. A pregnancy test was carried out on all female patients prior to therapy and participants were asked to use double contraceptives during the trial. Apparently, this study was the start of a long association between the investigators at AIIMS and the company, which apparently continued to supply thalidomide for trials conducted after 2002.^79^

In July of the same year, *The Times of India* reported a regulatory intervention of the government of India in this practice of direct procurement of thalidomide. The newspaper informed the public that an undertaking between the government and Grünenthal was signed after the company had stopped thalidomide supplies and insisted on making sure that ‘it would not to be held responsible in the event of any adverse reaction of the drug’. The corresponding undertaking signed in 1987 in respect to leprosy patients had put an end to the first interruption of supplies and designated the CLTRI as nodal centre for dispensing thalidomide for the declared purpose of treating leprosy reactions. This arrangement did not legally impede Grünenthal from providing the drug for the above-mentioned clinical trials on cancer. Ironically, however, by doing so the company itself had ignored its previous insistence on channelling thalidomide through one national institution and thereby avoiding any liability for its known adverse reactions. Conversely, in 2002 it was the government that explicitly asked Grünenthal to only supply the drug to the CLTRI so that it could be dispensed from there to the AIIMS for ‘experimental use’.^80^

The Central Drugs and Standard Control Organisation (CDSCO) decided to approve the first drug containing thalidomide in India only a few weeks later, on 18 August 2002, four years after its first licensing for ENL in the USA. It was licensed for ‘acute treatment of the cutaneous manifestation of moderate to severe erythema nodosum leprosum and for the treatment of multiple myeloma’.^81^ Some of more than forty thalidomide manufacturers that appeared on the Indian market until 2020 also exported substantial quantities to other parts of the world. On the home market, prohibitive costs and limited availability was still a concern in 2009, when it was estimated that a three-month dose of thalidomide cost 17 550 Indian rupees, which was unaffordable for most patients.^82^

Two analogues of thalidomide, lenalidomide and pomalidomide, have improved anti-inflammatory activity and are considered to be more effective. Their degree of embryotoxicity in humans is not known. Approval for lenalidomide was given in the USA in 2006. In India, the CDSCO licensed it for specific conditions of transfusion-dependent anaemia in 2007 and for treating multiple myeloma patients from 2008.^83^ Pomalidomide, a molecule that has a hybrid structure between lenalidomide and thalidomide, followed in 2013.^84^ There is an observable tendency towards further testing the utility of thalidomide and its analogues, as for instance in Hailey-Hailey disease and β-thalassaemia,^85^ thus increasing the global level of consumption. In India, the CLTRI and the National Leprosy Eradication Programme remains responsible for the distribution of thalidomide within the government health system. However, seventy per cent of the Indian population accesses healthcare from the private health sector through personal expenditure. Today, thalidomide can be prescribed by private practitioners and is widely available not only from local level pharmacies in India but can also be purchased through online pharmacies after uploading a prescription.

## Conclusion: the risk of thalidomide embryopathy

The available sources indicate that India’s industrialization and import substitution policy was a crucial condition for preventing the use of drugs containing thalidomide around 1960. In telling contrast, they were freely imported and available by prescription in the Portuguese territory of Goa before it was integrated into the Republic of India from December 1961. The limited quantities, however, may not have led to any births of persons affected by thalidomide embryopathy. After 1965, thalidomide was introduced to India through international networks of voluntary organizations that were supporting the official leprosy control programmes by providing medical care and shelter to leprosy patients. These NGOs were largely independent in using ‘investigational drugs’. Indian state health authorities depended on them since colonial times in providing modern leprosy therapy. The crucial importance of the NGOs only waned after the introduction of multidrug therapy in the 1980s and the subsequent decrease of leprosy patients.

Despite the free supplies of the German producer, the supply of thalidomide in India was marginal until its more widespread use from the 1970s. After being curbed by the distribution control of a central state institution between 1986 and its official approval in 2002, the scenario changed completely. In India the use of the drug has continuously increased just like in many other countries around the world. Globally, the number of thalidomide producers has grown considerably. During the last two decades, most manufacturers of thalidomide and its analogues, including those in India, have marketed these drugs as anti-oncology products. While the majority of cancer patients are beyond reproductive age, the tendency to approve additional indications for these drugs is posing new challenges. Like in other countries, the unresolved risk of prenatal damages has led to the implementation of regulatory measures, which contribute to reducing these incidences but cannot completely prevent them, as the Brazilian experience shows.^86^

This regulatory process commenced in the USA with the System for Thalidomide Education and Prescribing Safety (STEPS) that was approved in connection with licensing of the drug, Thalomid, in 1998. Since 2010 the prescription of thalidomide and its analogues has been regulated under the Risk Evaluation and Mitigation Strategy (REMS) of the FDA. The responsibility for implementing these schemes rests with the manufacturer.^87^ In Brazil the first law controlling the drug was enacted in 2003. Thalidomide is being produced by a state-owned pharmaceutical company and distributed through the Ministry of Health. In contrast, the government of India opened the market for many manufacturers by licensing the first drug containing thalidomide in August 2002 and then slowly adopting more stringent measures for reducing and monitoring the embryotoxic risk, particularly its guidelines for the use of thalidomide in the management of ENL (2012)^88^ and of thalidomide and lenalidomide for multiple myeloma (2017).^89^ Central elements of these regulatory provisions for ENL are a form for recording details of the patients upon thalidomide treatment and, in the case of women of childbearing age, a pregnancy test and counselling on double contraceptive measures. While these regulations are binding only for government institutions treating patients with ENL complications, the actual overall control is weak and the collection of data ineffective.

In contrast to Europe, the USA, Japan, Australia and Latin American countries, there is no birth defects surveillance in India that can provide an opportunity to detect the prenatal damages caused by embryotoxic drugs.^90^ Despite the availability of thalidomide in India, publications spanning the period from the 1960s onwards have not reported any unusual increase in the numbers of typical limb deformity cases.^91^ The earliest reported effort in identifying thalidomide-induced anomalies was apparently made in 1969 by Dr Charles Pinto, K.E.M. Hospital and G.S. Medical College, Bombay, who observed a ‘case of cleft lip […] in connection with intake of thalidomide’.^92^ The result of this investigation is not documented. In 2013, newspapers reported on an Indian student with shortened and deformed limbs who claimed maternal use of thalidomide in pregnancy as antiemetic. In connection with a lawsuit filed by this student, the Bombay High Court asked the drug administration to consider issuing a circular for doctors, pointing out the side effects of thalidomide.^93^ Without any clinical and genetic investigations, it cannot be established whether reports of this kind refer to true cases of thalidomide embryopathy.

Up to the official approval of thalidomide in 2002, the embryotoxic risk mainly depended on the safety measures taken by prescribing doctors and patients. A secondary responsibility rested with the thalidomide supplier, which protected itself against any liability by making thalidomide recipients sign corresponding declarations from the mid-1970s and requiring the government of India to establish a national distribution center ten years later. The critical attitude of its physicians, conforming with the reserved position of the government and the WHO, halted the further spread of thalidomide as a remedy for leprosy reactions. The embryotoxic risk under these conditions was low. Whether there was a very limited number of thalidomide embryopathy cases, however, may never be known given the lack of surveillance for birth defects. The increasing use of thalidomide and other embryotoxic drugs during the last two decades poses new challenges. The approval of these active substances has shifted a greater amount of social and legal responsibility to the government of India and other public institutions.

Even though compensation claims for prenatal drug damages have not yet been reported in India, it is not unlikely that well-founded demands will be brought forward in the future. Internationally, there are no general standards for legal accountability and other provisions to tackle this problem. The difficulties of dealing with thalidomide-induced birth defects can be seen in the case of Spain where some of these were officially recognized only after more than fifty years when there was virtually no evidence left for the intake of the drug by the mothers. As the claims of the affected persons against the thalidomide producers were time-barred, they are receiving payments by the state. In Australia, a court decided in favour of the claimants who now get compensation from the company concerned.^94^ Holding the pharmaceutical industry accountable and limiting the embryotoxic risk in India would require it to introduce a monitoring system for the private sector, which may be similar to the one practiced in government hospitals. Adopting the more elaborate safety standards of industrialized countries, and with it accepting the high prices for thalidomide and its analogues, does not appear to be a realistic option in the foreseeable future. The more limited resources have led to a specific but also unsatisfactory regulatory compromise. Under these conditions, it will be necessary to strike a different balance between the conflicting interests of patients, producers and the state in India.

## Footnotes

1 N. Vargesson, ‘Thalidomide‐induced Teratogenesis: History and Mechanisms,’ *Birth Defects Research*, 100, Part C (2015), 140–56.

2

S. Mansour , E. Baple and C.M. Hall , ‘A Clinical Review and Introduction of the Diagnostic Algorithm for Thalidomide Embryopathy (DATE)’, Journal of Hand Surgery (European Volume), 44, 1 (2019), 96–108.3025368510.1177/1753193418800631

3 When it was firstly approved for this indication in the USA in 1998, it was being used for other indications, too, cf. S. Timmermans and V. Leiter , ‘The Redemption of Thalidomide: Standardizing the Risk of Birth Defects’, Social Studies of Science, 30, 1 (2000), 41–71 1162467910.1177/030631200030001002.

4 In English e.g. Henning Sjöström and Robert Nilsson, *Thalidomide and the Power of the Drug Companies* (Harmondsworth: Penguin, 1972); Widukind Lenz, ‘A Short History of Thalidomide Embryopathy’, *Teratology*, 38, 3 (1988), 203–215; Nadja Yllner, *Just a Little White Sleeping Pill: The Story of the Neurosedyn Catastrophe* (Borås: Recito, 2008).

5 An exception to this is a historical study that covers five countries: Julie Parle and Ludger Wimmelbücker , ‘These are the medicines that “make” monsters’: Thalidomide in Southern Africa, 1958–1962’, Social History of Medicine, 33, 3 (2020), 898–923 3272831210.1093/shm/hkz011PMC7375878.

6 The child mortality is usually estimated at 40 per cent, cf. Lenz, *op. cit.* (note 4), 205.

7 Accessible at the State Archives of North-Rhine Westphalia, Rhineland section (Landesarchiv Nordrhein-Westfalen, Abteilungen Rheinland, LAV NRW R, Gerichte Rep. 139), in the following cited as LAV NRW.

8 Israel State Archives, Jerusalem, hereafter cited as ISA.

9 Monthly report of the commercial management for October 1954, 22 November 1954, January 1956, 24 February 1956, April 1956, 17 May 1956, September 1956 and 19 October 1956. LAV NRW, no. 169, f. 146; no. 170, f. 314; no. 170, f. 132; no. 177, f. 977; monthly report of the foreign department for June 1957, 10 July 1957, no. 177, f. 23.

10 Government of India, Patent Office, specification no. 56475, applied for on 3 February 1956, accepted on 26 November 1957. *Noctosediv* and *Softenon* were probably not the only trademarks registered in India, cf. the monthly reports of the trademarks department for May 1959 and April 1961. LAV NRW, no. 180, f. 312, and no. 234, f. 928.

11 Monthly report of the foreign department, 6 March 1961. LAV NRW, no. 234, f. 616; cf. the official trips of Dr Günter Nowel, no. 12, f. 362–70.

12 Monthly reports of the foreign department for December 1956, 10 January 1957, November 1957, 10 December 1957, January 1958, 11 February 1958, July 1958, 8 August 1958. LAV NRW, no. 177, f. 666; no. 178, f. 440; no. 178, f. 676–677; no. 179, f. 153.

13 Monthly report of the foreign department for July 1959, 10 August 1959, September 1959, 7 October 1959, November 1959, 10 December 1959, July 1960, 9 August 1960, etc. LAV NRW, no. 180, f. 409; no. 180, f. 637; no. 180, f. 887–888; no. 181, f. 812.

14 Monthly report of the licence department for July 1961, 10 August 1961. LAV NRW, no. 238, f. 618.

15 Memorandum of the talks at DCBL from 25–27 January 1961, dated 1 February 1961. LAV NRW, no. 249, f. 556.

16 ‘Draft of the Drugs (Amendment) Rules, 1962’, *The Gazette of India*, no. 30, 28 July 1962, 2648 and 2651.

17 ‘Deformed Babies Born’, *The Tribune*, 25 August 1962, 4; ‘Ban on Import of Thalidomide’, *The Pioneer*, 25 August 1962, 4.

18 ‘Thalidomide Jeopardises 5000 Babies’, *Journal of the Indian Medical Association*, vol. 39, no. 5, 1 September 1962, 270; cf. the article with the same title in *The Statesman*, 18 July 1962, 6.

19 ‘Unsafe Sedatives on Sale in 2 Cities’ *Times of India*, 15 September 1962, 1; ‘Govt. Ban on Thalidomide’, *Times of India*, 16 September 1962, 1. The article on the press note was reprinted in the *Indian Journal of Medical Sciences*, vol. 16, no. 11, November 1962, VII.

20 ‘Dangerous Drug’, *Times of India*, 17 September 1962, 6.

21 LAV NRW, no. 14, f. 19–20.

22 LAV NRW, *ibid.* f. 19.

23 The distribution of around 10kg of thalidomide correspond to one surviving case of thalidomide embryopathy in Germany according to the figures of Lenz, *op. cit.* (note 4), 208.

24

J. Sheskin , ‘Thalidomide in the Treatment of Lepra Reactions’, Clinical Pharmacology & Therapeutics, 6, 3 (1965), 303–061429602710.1002/cpt196563303.

25 Perpétua do Socorro Silva Costa *et al.*, ‘Erythema Nodosum Leprosum: Update and Challenges on the Treatment of a Neglected Condition’, *Acta Tropica*, 183 (2018), 134–41. There is wide variation in the data on the number of patients who develop ENL, but leprosy patients with high bacillary load (cases of lepromatous leprosy) are at a higher risk of developing ENL.

26

L.M. Bechelli and V. Martínez Domínguez , ‘The Leprosy Problem in the World’, Bulletin of the World Health Organization, 34, 6 (1966), 817 PMC24760395296533; S.K. Noordeen , L. Lopez Bravo and T.K Sundaresan , ‘Estimated Number of Leprosy Cases in the World’, Bulletin of the World Health Organization, 70, 1 (1992), 9PMC23933561568282.

27 A combination of rifampicin, clofazimine and dapsone for multibacillary cases (patients with a high bacillary load) and rifampicin and dapsone for paucibacillary (low bacillary load) cases. The WHO has officially recommended MDT for the treatment of leprosy since 1982.

28

C.G. Voorend and E.B. Post analysed studies published after 1980: ‘A Systematic Review on the Epidemiological Data of Erythema Nodosum Leprosum, a Type 2 Leprosy Reaction’, PLoS neglected tropical diseases, 7, 10 (2013), e2440 2409881910.1371/journal.pntd.0002440PMC3789767; P.S. Rao , M. Subramanian and G. Subramanian , ‘Deformity Incidence in Leprosy Patients Treated with Multidrug Therapy’, Indian Journal of Leprosy, 66, 4 (1994), 449–547714354.

29

J. Parascandola , ‘Chaulmoogra Oil and the Treatment of Leprosy’, Pharmacy in History, 45, 2 (2003), 47–56 12894769.; F. S. D. D. Santos , L.P.A.D. Souza and A.C. Siani , ‘Chaulmoogra Oil as Scientific Knowledge: The Construction of a Treatment for Leprosy’, História, Ciências, Saúde-Manguinhos, 15, 1 (2008), 29–46 1924167210.1590/s0104-59702008000100003.

30 Dr Dharmendra, ‘Leprosy in Ancient Indian Medicine’, *International Journal of Leprosy*, 15 (1947), 424–430. The texts referred to a treatment of leprosy using ‘tuvarka’, a seed that could be taken either orally or via oil extracts that could be applied topically onto the skin. Although Dharmendra had some reservations about the identity of this seed, most scholars identify this as chaulmoogra.

31

A.B. Karat and K. Ramanujam , ‘A Century of Progress in the Therapy of Leprosy’, International Journal of Leprosy, 41, 3 (1973), 382–914130572.

32

J. T. Jacob and C. Franco-Paredes , ‘The Stigmatization of Leprosy in India and its Impact on Future Approaches to Elimination and Control,’ PLoS Neglected Tropical Diseases, 2, 1 (2008), e113 1823584510.1371/journal.pntd.0000113PMC2217676.

33 S. Patil *et al.*, ‘Towards Elimination of Stigma & Untouchability: A Case for Leprosy,’ *The Indian Journal of Medical Research*, 149, suppl. 1, (2019), S81–7.

34 Response of Sagher to a letter of Sister Elli, DAHW, 27 March 1969; Dr Marcel Farine, the president of Swiss Emmaus, to Sagher, 30 November 1965. ISA, GL-53396/3 and GL-53426/10, n.p.

35 Sagher to Shyam Deshpande in Amravati, 27 September 1966, and the response dated 12 October 1966. ISA, GL-53426/10, n.p.

36 Dastur to Sagher, 14 November 1966; Divekar to Sagher, 15 November 1966; Sagher to Divekar, 30 December 1966. ISA, GL-53426/10, n.p.; cf. D.K. Dastur, S.C. Divekar and N.H. Antia, ‘Leprous Neuritis of Nerve Trunks’, *International Journal of Leprosy*, 36, 4 (1968), part 2, 622–623; A. Magora, J. Sheskin and F. Sagher, ‘Motor Conduction Velocity Test of the Ulnar Nerve in Leprosy Reaction under Thalidomide Therapy’, *ibid.* 624.

37 Sagher to Antia, 5 July 1966; Antia to Sagher, 10 November 1966; Sagher to S.C. Desai, 6 September 1966; Desai to Sagher, 26 August 1966; Dr Mannheim, Grünenthal, to Sagher, undated telegram among the letters of 1966, carrying a stamp of 2 March 1967. ISA, GL-53426/10, n.p; cf. J. Sheskin *et al.*, ‘Unsatisfactory Results with Thalidomide as a Specific Treatment for Leprosy’, *Israel Journal of Medical Sciences*, 4, 4 (1968), 904, note 13.

38 Von Schrader-Beielstein to Wintsch, 8 February 1967. ISA, GL-53426/8, n.p. The husband of Lena Wintsch-Koller (1930–2015), Jacob (Kob) Wintsch, worked as surgeon at the same hospital.

39 Wintsch to von Schrader-Beielstein, 17 April 1967. ISA, GL-53426/8, n.p.

40 Wintsch to Sagher, 29 November 1968 and 19 December 1972. ISA, GL-53396-4 and GL-53426/8, n.p.

41 Valentine Patrick Macaden to von Schrader, 22 August 1968, and von Schrader to Emmaus, 3 September 1968, ISA, GL-53401/15, n.p.

42 Von Schrader to Rebello, 3 December 1967; Rebello to Grünenthal, 24 January 1968; von Schrader to Rebello and to the Drugs Controller, New Delhi, both letters dated 29 January 1968; Rebello, to Sagher, 10 February 1968. ISA, GL-53426/6, n.p.

43 List of persons who did not answer the questionnaire of Sheskin and Sagher: Fragebogen über Thalidomid-Behandlung bei Lepra-Reaktion, C) Liste von ausgesandten nicht beantworteten Fragebögen, page 6. ISA, GL-53396/8, n.p. In a letter to Sagher she expressed her wish to visit Venezuela to learn more about the use of thalidomide and to participate in a meeting on thalidomide in London, but apparently lacked both the necessary travel funds and professional contacts, Rebello to Sagher, 1 December 1967. ISA, GL-53426/6, n.p.

44 A.B.A. Karat to Helen Taussig, 8 November 1967. Alan Mason Chesney Medical Archives, Johns Hopkins University, Baltimore, Helen B. Taussig Collection, F 38/18.

45 CLTRI website at https://cltri.gov.in/about/default.html (accessed on 16 June 2021).

46 Von Schrader to Iyer, 16 January 1967, ISA, GL-53426/10, n.p. According to Iyer thalidomide had been used only for the below-mentioned trial, cf. Iyer to Sagher 21 November 1972, ISA, GL-53396/8, n.p.

47

C.G.S. Iyer et al., ‘WHO Co-ordinated Short-term Double-blind Trial with Thalidomide in the Treatment of Acute Lepra Reactions in Male Lepromatous Patients’, Bulletin of the World Health Organization, 45, 6 (1971), 731 PMC24279774947831.

48 List of negative responses to the questionnaire of Sheskin and Sagher: Fragebogen über Thalidomid-Behandlung bei Lepra-Reaktion, B) Liste von negativen Antworten, page 2. ISA, GL-53396/8, n.p.

49 Vomstein to Sheskin, 24 December 1972, enclosed: Covering Report and the completed questionnaire. ISA, GL-53396/9, n.p.

50 Completed questionnaire, 30 December 1972. ISA, GL-53396/9, n.p.

51 They were part of a more extensive study of Isoprodian led by Enno Freerksen, cf. E. Freerksen, S.G. Browne and J.H. Thumin (eds), ‘The Chemotherapy of Leprosy Today and Tomorrow: Second International Leprosy Colloquium, held at the Forschungsinstitut Borstel, October 15 and 16, 1974’, *Leprosy Review*, 46, 2 (1975), suppl.

52

E. Vomstein , ‘Preliminary Report of a Drug Trial Conducted at Leprosy Relief Rural Centre, Chettipatty, South India’, Leprosy Review, 46, 2 (1975), suppl., 207–13110096210.5935/0305-7518.19750067.

53

M. Aschhoff , ‘Treatment of Leprosy with Rifampicin and Isoprodian in 38 patients at St. Thomas Hospital, Chetput, South India’; Leprosy Review, 46, 2 (1975), suppl., 173–78116569710.5935/0305-7518.19750062, 174–5 (quotations).

54 Adressenliste CG-217 (Thalidomid). ISA, GL-53396/9, n.p.

55 See the contracts and the progress reports in WHO archives, L4/181/14, and the publications: K. Ramanujam, C.G.S. Iyer and G. Ramu, ‘Open Trial with Clofazimine in the Management of Recurrent Lepra Reaction and of Sulphone Sensitive Cases: A Preliminary Report’, *Leprosy in India*, 46 (1975), suppl., 117–20; C.G.S. Iyer and G. Ramu, ‘An Open Trial with Clofazimine in the Management of Recurrent Lepra Reaction Using Thalidomide as a Control Drug’, *Leprosy in India*, 48, 4 (1976), suppl., 690–4; G. Ramu and A. Girdhar, ‘Treatment of Steroid Dependent Cases of Recurrent Lepra Reaction with a Combination of Thalidomide and Clofazimine’, *Leprosy in India*, 51, 4 (1979), 497–504.

56 The Leprosy Division at WHO headquarters to Communicable Disease Surveillance (CDS) advisor, South-East Asia Regional Office (SEARO), Re. Supply of thalidomide tablets, 10 October 1975. WHO archives, IND-BVD-001 LEP 8, n.p; cf. the covering letter (without the attached declaration): Iyer to the Chief Medical Officer, Leprosy, H. Sansarricq, 11 July 1975. WHO archives, L4/446/T-12/3, n.p.

57 S. Theophilus, ‘Treatment with Thalidomide in Steroid Dependency and Neuritis’, *Leprosy in India*, 52/3 (1980), 423–28; M.A. Rajan, M.S. Rajagopalan and M. Joseph, ‘A Clinical Study of Thalidomide comparing Pre-treatment and Post-treatment Reactional Episodes and Corticosteroid Requirements’, *Leprosy in India*, 55, l (1983), 111–16; D.A. Parikh, R. Ganapati and C.R. Revankar, ‘Thalidomide in Leprosy – Study of 94 Cases’, *Indian Journal of Leprosy*, 58, 4 (1986), 565 (quotation).

58

A.G. Chandorkar et al., ‘Thalidomide in Lepra Reaction (ENL) in Lepromatous Leprosy Patients’, Indian Journal of Leprosy, 56, 2 (1984), 264–686548496; V.H. Jadhav , A.H. Patki and J.M. Mehta , ‘Thalidomide in Type-2 Lepra Reaction – A Clinical Experience’, Indian Journal of Leprosy, 63, 3 (1990), 316–202262716.

59 As reported in the publications. The ‘fourtynine adult patients’ who participated in the trial of Rajan *et al.* (1983) were possibly male, too. Some of the reported 246 test persons probably participated in more than one trial.

60 G. Ramu and C.G.S. Iyer, ‘Treatment of Reactions in leprosy’, in B.R. Chatterjee (ed.), *A Window on Leprosy* (Wardha: Gandhi Memorial Leprosy Foundation, 1978), 256.

61 WHO Expert Committee on Leprosy. Forth Report (Geneva: WHO, 1970), 23.

62 Grünenthal, List of thalidomide requests since August 1984 attached to a letter dated 1 February 1985, WHO archives, L4/447/5, vol. 1, n.p.

63 SEARO to Leprosy Division at WHO headquarters, Re. Supply of thalidomide, 2 July 1985, and attached the signed ‘Certification’ for Grünenthal dated 1 June 1985. WHO archives, L4/447/5, vol. 2, n.p.

64 See the photocopy of an import licence for 20 000 tablets for the Vimala Dermatology Centre, Bombay, attached to the letter of Dr Liliane Migliavacca to S.K. Noordeen, 4 February 1985. WHO archives, L4/447/5, vol. 1, n.p.

65 As mentioned in the letter of A. Dutta to the medical director of Grünenthal, 11 April 1991. WHO archives, L4/447/5, vol. 3, n.p.

66 C.K. Rao, Deputy Director of Health Services, to N.K. Shah, the Senior Public Health Administrator of the WHO in New Delhi, 2 September 1986. WHO archives, L4/447/5, vol. 2, n.p.

67 A. Dutta, the director of CLTRI, to Grünenthal, 11 April 1991. WHO archives, L4/447/5, vol. 3, n.p.

68 B.N. Mittal, Director of Health Services, to K. Wilsmann, medical director of Grünenthal, 22 April 1991 and 26. May 1993. WHO archives, L4/447/5, vol. 3, n.p.

69 R. Ganapati, the vice-president, to the president, K.V. Desikan, 5 April 1985, attached to Ganapati to Noordeen, 15 May 1985. WHO archives, L4/447/5, vol. 1, n.p. Dr Ganapati was director of the Bombay Leprosy Project and headed the Central JALMA Institute for Leprosy, Agra, originally established by the Japanese Leprosy Mission for Asia (JALMA), a voluntary organization based in Tokyo, and taken over by the government in 1976.

70 Metha to Reiza de Nordeste, São Paulo, 3 February 1987; Metha to S.K. Noordeen, Chief Medical Officer, Leprosy Division, WHO, 8 August 1991. WHO Archives, L4/447/5, vol. 2, n.p.

71 V.H. Jadhav, personal communication, November 2020. Dr Jadhav said that during his tenure as Chief Medical Officer at Bandorwallah Leprosy Hospital, Pune, thalidomide supplies were erratic. But the leprosy hospital in Pune procured thalidomide from DAHW, which at some time had ordered thalidomide from Brazil.

72 Dr José Terencio de las Aguas donated 500 tablets on his visit to the project and subsequently sent another consignment to Dr C.R. Revankar, which was limited as the Health Ministry in Spain strictly controlled the distribution of thalidomide, cf. Revankar to Terencio de las Aguas, 21 May 1991, and Terencio de las Aguas to Revankar, 20 July 1991. Archivo de la Diputación Provincial de Alicante, Alicante, Archivo del Sanatorio de Fontilles, box 95. Thalidomide was used at the Bombay Leprosy Project already before 1985, even in a clinical trial, cf. Parikh, Ganapati and Revankar, *op. cit.* (note 57).

73 Only two studies from India were reported during this period: Jadhav, Patki and Mehta, *op. cit.* (note 58); V. Kishore, ‘Thalidomide – A Clinical Study of 33 Cases of Type II Lepra Reaction’, *International Leprosy Congress. Abstracts. Orlando, Florida, August 29 thru September 4, 1993* (London: IELP), 23A.

74 L. Pocaterra *et al.*, ‘Clinical Course of Erythema Nodosum Leprosum: An 11-year Cohort Study in Hyderabad, India’, *The American Journal of Tropical Medicine and Hygiene*, 74, 5 (2006), 868–79, see 878; cf. A. Girdhar, J.K. Chakma and B.K. Girdhar, ‘Pulsed Corticosteroid Therapy in Patients with Chronic Recurrent ENL’, *Indian Journal of Leprosy*, 74, 3 (2002), 233–36; B.R. Kar and R. Babu, ‘Methotrexate in Resistant ENL’, *International Journal of Leprosy*, 72, 4 (2004), 10–12.

75 See e.g. J.K. Grover et al., ‘Thalidomide: A Re-look’, The National Medical Journal of India, 13, 3 (2000), 132–4111558112.

76

R.J. D’Amato et al. ‘Thalidomide is an Inhibitor of Angiogenesis’, Proceedings of the National Academy of Sciences, 91, 9 (1994), 4082–510.1073/pnas.91.9.4082PMC437277513432.

77

S. Singhal et al., ‘Antitumor Activity of Thalidomide in Refractory Multiple Myeloma’, New England Journal of Medicine, 341, 21 (1999), 1565–711056468510.1056/NEJM199911183412102.

78

J.K. Grover , G. Uppal and V. Raina , ‘The Adverse Effects of Thalidomide in Relapsed and Refractory Patients of Multiple Myeloma’, Annals of Oncology, 13, 10 (2002), 1636–401237765410.1093/annonc/mdf258.

79

A. Sharma et al., ‘Long Term Use of Thalidomide: Safe and Effective’, Indian Journal of Cancer, 44, 3 (2007), 108–091825053210.4103/0019-509x.38942; G. Uppal et al., ‘Use of Simple Hematological, Biochemical and Clinical Parameters to Monitor Response of Multiple Myeloma Patients on High Dose Thalidomide Therapy’, Indian Journal of Cancer, 42, 1 (2005), 42–610.4103/0019-509x.1510015805692.

80 ‘Supply of Cancer Drug Thalidomide Resumed’, *The Times of India*, 30 July 2002, https://timesofindia.indiatimes.com/city/delhi/Supply-of-cancer-drug-Thalidomide-resumed/articleshow/17498672.cms (accessed on 16 June 2022).

81 No. 3535, Thalidomide caps (50 and 100mg). https://cdscoonline.gov.in/CDSCO/Drugs (accessed on 16 June 2022).

82

A. Palit , ‘Management of Leprosy Reactions: Facing the Realities’, Indian Journal of Leprosy, 81, 4 (2009), 238–9.

83 Nos. 3235 and 348, *op. cit.* (note 81).

84 A. Jandial *et al.*, ‘Real World Experience with “Generic” Pomalidomide in Relapsed Refractory Multiple Myeloma’, *Leukemia & Lymphoma*, 60, 4 (2019), 1102–04.

85 For trials in India see e.g. K.B. Nanda et al., ‘Hailey-Hailey Disease Responding to Thalidomide’, Indian Journal of Dermatology, 59, 2 (2014), 190–922470094110.4103/0019-5154.127684PMC3969682; J. Chandra et al., ‘Efficacy and Safety of Thalidomide in Patients with Transfusion-dependent Thalassemia’, Indian Pediatrics, 58 (2021), 611–1634315832.

86

F.S.L. Vianna et al., ‘The Impact of Thalidomide Use in Birth Defects in Brazil’, European Journal of Medical Genetics, 60, 1 (2017), 12–15 2763833010.1016/j.ejmg.2016.09.015.

87 For the Thalomid® (thalidomide) REMS provisions see https://www.accessdata.fda.gov/drugsatfda_docs/rems/Thalomid_2021_08_05_REMS_Full.pdf (accessed on 16 June 2022).

88 Central Leprosy Division, Directorate General of Health Services. Disabiltiy Prevention and Medical Rehabilitation: Guidelines for Primary, Secondary and Tertiary Level Care. http://www.clinicalestablishments.gov.in/WriteReadData/516.pdf (accessed on 16 June 2022).

89 Indian Council of Medical Research. Consensus Document for Management of Multiple Myeloma. https://main.icmr.nic.in/sites/default/files/guidelines/Multiple%20Myeloma_0.pdf (accessed on 16 June 2022). The document points out that the risk of peripheral neuropathy and thrombosis limits the use of thalidomide in maintenance treatment.

90 P. Bhide, ‘Birth Defects Surveillance in India’, in A. Kar (ed.), *Birth Defects in India: Epidemiology and Public Health Implications* (Singapore: Springer, 2021), 97–115.

91 A. Kar and L. Wimmelbücker, ‘Thalidomide in India’, manuscript.

92 Lenz to Pinto, 14 January 1969. Archives of the University of Münster, Germany, Papers of Widukind Lenz, no. 541, f. 151.

93 ‘Thalidomide Teen Can Do His MBBS Course, Says High Court’, *The Times of India*, 7 September 2013, 6; ‘Consider Issuing Circular on Side-effects of Drug “Thalidomide”: Bombay High Court to FDA’, *The Economic Times*, online edition (accessed on 16 June 2022).

94 See e.g. James Badcock, ‘Spain’s Forgotten Thalidomide Victims see Glimmer of Hope’, *BBC News*, 23 December 2016, https://www.bbc.com/news/world-38386021 (accessed on 16 June 2022); Michael Magazanik, *Silent Shock: The Men Behind the Thalidomide Scandal and an Australian Family’s Long Road to Justice* (Melbourne: The Text Publishing Co., 2015).

